# Predicting the Survival of Patients With Cancer From Their Initial Oncology Consultation Document Using Natural Language Processing

**DOI:** 10.1001/jamanetworkopen.2023.0813

**Published:** 2023-02-27

**Authors:** John-Jose Nunez, Bonnie Leung, Cheryl Ho, Alan T. Bates, Raymond T. Ng

**Affiliations:** 1 BC Cancer, Vancouver, British Columbia, Canada; 2 Department of Computer Science, University of British Columbia, Vancouver, British Columbia, Canada; 3 Department of Psychiatry, University of British Columbia, Vancouver, British Columbia, Canada

## Abstract

**Importance:**

Predicting short- and long-term survival of patients with cancer may improve their care. Prior predictive models either use data with limited availability or predict the outcome of only 1 type of cancer.

**Objective:**

To investigate whether natural language processing can predict survival of patients with general cancer from a patient’s initial oncologist consultation document.

**Design, Setting, and Participants:**

This retrospective prognostic study used data from 47 625 of 59 800 patients who started cancer care at any of the 6 BC Cancer sites located in the province of British Columbia between April 1, 2011, and December 31, 2016. Mortality data were updated until April 6, 2022, and data were analyzed from update until September 30, 2022. All patients with a medical or radiation oncologist consultation document generated within 180 days of diagnosis were included; patients seen for multiple cancers were excluded.

**Exposures:**

Initial oncologist consultation documents were analyzed using traditional and neural language models.

**Main Outcomes and Measures:**

The primary outcome was the performance of the predictive models, including balanced accuracy and receiver operating characteristics area under the curve (AUC). The secondary outcome was investigating what words the models used.

**Results:**

Of the 47 625 patients in the sample, 25 428 (53.4%) were female and 22 197 (46.6%) were male, with a mean (SD) age of 64.9 (13.7) years. A total of 41 447 patients (87.0%) survived 6 months, 31 143 (65.4%) survived 36 months, and 27 880 (58.5%) survived 60 months, calculated from their initial oncologist consultation. The best models achieved a balanced accuracy of 0.856 (AUC, 0.928) for predicting 6-month survival, 0.842 (AUC, 0.918) for 36-month survival, and 0.837 (AUC, 0.918) for 60-month survival, on a holdout test set. Differences in what words were important for predicting 6- vs 60-month survival were found.

**Conclusions and Relevance:**

These findings suggest that models performed comparably with or better than previous models predicting cancer survival and that they may be able to predict survival using readily available data without focusing on 1 cancer type.

## Key Points

**Question:**

Can natural language processing be used to predict the survival of patients with cancer from their initial oncologist consultation document?

**Findings:**

In this prognostic study of 47 625 patients with cancer, 6-, 36-, and 60-month survival were predicted using both traditional and neural language models with performance similar to or better than that found in prior work.

**Meaning:**

These findings suggest it is feasible to predict survival of patients with cancer using a common cancer document without additional data and without training separate models for specific types of cancer.

## Teaser

This prognostic study develops and evaluates neural natural language processing models that predict the survival of patients with general cancer using their initial oncologist consultation.

## Introduction

Cancer is a leading cause of death globally, with survival depending on variables including cancer site, tumor type, age, sex, and comorbidities.^1^ Accurately predicting an individual patient’s survival could be used to improve cancer care. For example, it might suggest earlier referral to palliative care resources or consideration of more aggressive therapies upfront. Traditionally, survival rates are calculated retrospectively and categorized by only a few factors, primarily by cancer site and histology.^1^ Despite familiarity with these odds, oncologists can be inaccurate when predicting an individual patient’s survival prospectively^2^; they have trouble factoring in personal factors such as age.

Predictive models trained by machine learning may allow more personalized predictions by using many features of a patient’s particular characteristics and disease, and have been shown to outperform the prediction of treating oncologists.^3^ Some models developed to date utilize structured data, that is, data that are processed into specific features such as the presence of genetic markers, demographics, or specific aspects of clinical history.^4,5,6,7,8^ This may limit the widespread use of such models, as data availability varies among cancer treatment centers and between patients. It also limits what data can be used for a model, as not all clinical data are easily coded or categorized for extraction and analysis.^9^ The use of unstructured data, such as text within medical documents, may address these drawbacks. Almost all patients receiving treatment for cancer have an initial consultation document from their oncologist. Such documents generally have many details relevant to survival, for example, tobacco use or marital status, even if the clinic does not routinely store such data in structured data sets.

The use of machine learning with documents, known as natural language processing (NLP), has increasingly been applied throughout medicine.^10,11,12^ Many of the applications both in medicine generally and cancer specifically have utilized smaller, specific documents such as radiology or pathology reports.^13,14^ Some studies such as that by Liu et al^15^ have used patient encounter documents to predict the onset of 3 noncancer illnesses. They found that models using the unstructured text data in these documents outperformed using only structured data, and that adding structured data like demographics and laboratory data increased performance by only a marginal amount. Within cancer, recent work predicted survival in patients with lung cancer by extracting structured data from unstructured document data^16^ and used nonneural NLP on progress notes to update an individual’s prognosis.^17^

Neural networks are a type of machine learning modeled after the interconnectedness of neurons. When utilized in NLP, neural models can develop a more complex understanding of language, such as the presence of words with respect to each other, even when not adjacent.^4^ We were unable to find prior work using neural NLP methods to predict the survival of patients with general cancer using oncologist consultation documents, nor were such works identified in recent reviews of neural network applications in cancer,^13^ in general medical applications,^11^ or in a recent review of machine learning techniques used in cancer survival prediction.^18^

Our work sought to develop and evaluate neural NLP models that predict the survival of patients with general cancer using their initial oncologist consultation, without the use of structured data. By using this common document without structured data, we hoped to build models that would not be constrained by requiring the collection and processing of specific data. Similarly, we utilized our models with the patient population with general cancer seen across a provincial cancer control system, as opposed to focusing on predicting survival in patients with 1 cancer location treated in a single center. This allowed us to investigate the performance of more generalizable models. We also did not utilize any feature extraction techniques for our neural methods, instead allowing the neural networks to directly utilize the text after only low-level text preparation. We further sought to contribute to the field by comparing 2 common neural networks used in NLP with a recently developed neural network using transformers.^19^ We hypothesized that our models would be able to achieve predictive performance at least in line with prior work, predicting with an accuracy, balanced accuracy (BAC), and receiver operating characteristics area under the curve (AUC) all above 0.800 when tested upon a never-seen holdout set, and that the neural models would outperform the traditional nonneural bag-of-words (BoW) algorithm, with the models using transformers the best performing.

## Methods

The University of British Columbia BC Cancer Research Ethics Board approved this prognostic study, including exemption from requiring informed consent from study participants due to this being infeasible. We report this study following the Transparent Reporting of a Multivariable Prediction Model for Individual Prognosis or Diagnosis (TRIPOD) reporting guideline.

### Data Source

Our study cohort was selected from the 59 800 patients who started cancer care at BC Cancer between April 1, 2011, and December 30, 2016. These patients sought care for malignant disease or for precancerous or nonmalignant pathological findings that required specialist cancer care. BC Cancer provides all radiation therapy in British Columbia, and more than 85% of medical oncologists in the province have affiliation with the organization. BC Cancer cares for patients with cancer at 6 centers located in geographically diverse settings throughout the province and oversees systemic therapy at most of the 53 smaller Community Oncology Network sites. Data were provided to us by BC Cancer, which obtained mortality data from BC Vital Statistics administrative data.

### Data Selection and Preparation

We excluded patients who had more than 1 cancer diagnosis. We included all patients who had at least 1 valid document recorded as being a medical or radiation oncologist consultation generated within 180 days of diagnosis irrespective of disease and selected the document closest to the date of diagnosis. At BC Cancer, some patients may not see an oncologist for a few months after diagnosis as they may first receive surgery elsewhere, and come to the organization for chemotherapy, radiation therapy, or other specialized treatments.

We applied some preprocessing to documents before they were used by our models, as outlined in eMethods in Supplement 1. When used with BoW models, words had their endings removed to become tokens. We calculated survival as the number of months between the selected document being generated, and either the patient’s recorded death date or April 6, 2022, when mortality data were last extracted from administrative data. We then produced binary labels for whether a patient survived 6, 36, or 60 months or not.

### Natural Language Models

Language models are used in NLP to assign probabilities to the order of words,^20^ such as determining whether one sequence of words is more likely than another. We can extend this understanding to other tasks, such as predicting the binary survival outcomes in this work. Herein, we compare 4 language models: the traditional nonneural method, BoW,^21,22^ and 3 models using neural networks, consisting of convolutional neural networks (CNN),^23,24,25^ long short-term memory (LSTM),^26^ and a more recent transformer model, bidirectional encoder representations from transformers (BERT).^19^ We show simplified diagrams of these models in the Figure. Neural networks allow models to have more complicated understandings of language, such as how nearby or even distant words can change each other’s meaning. We provide further description of these models, including hyperparameters, in the eMethods in Supplement 1. We implemented our models in the Python 3 programming language. We describe further details, including libraries used^27,28,29,30^ and techniques for dealing with imbalance between survivors and nonsurvivors, in the eMethods in Supplement 1. Our code and trained models are publicly available through a GitHub repository.

**Figure.**
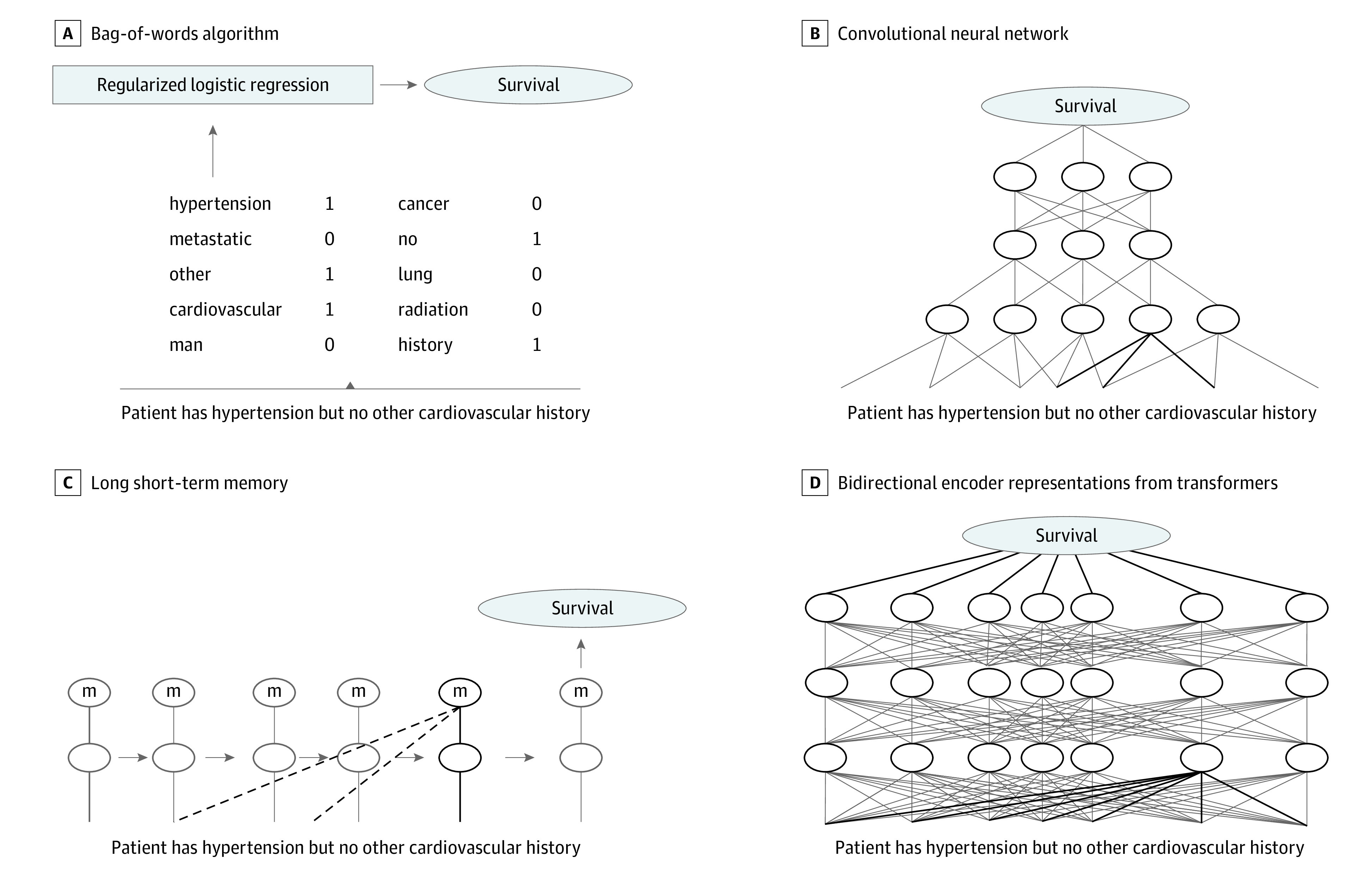
Simplified Diagrams of the Language Models Used in This Work A, The bag-of-words model counts word occurrences in a document, which is then used by a traditional machine learning algorithm. B, The convolutional neural network model understands a document in small adjacent clusters of words called convolutions (one is shown with black lines). The model can then learn to predict from combinations of these convolutions. C, The long short-term memory model updates the prediction by reading the document one word at a time. It has a memory cell that allows it to remember some prior context (dotted lines). D, The bidirectional encoder representations from transformers model can understand how each word is connected to all other words in the document but can only read small portions of text. One word’s possible connections are indicated by a black line.

### Statistical Analysis

The primary outcome was model performance when predicting patients’ survival of 6, 36, or 60 months. To avoid overfitting, when a model performed well on its training data but not on new data,^31^ we randomly separated our data into training (70%), development (10%) and testing (20%) sets. We trained our neural models on the training data up to 100 times (epochs), stopping when performance evaluated on the development set did not increase for 5 epochs. We used the training and development sets to tune our model hyperparameters, then evaluated the model on the test set to ensure no tuning or development could overfit the test set. We assessed model performance by reporting the accuracy, BAC, AUC, F1 score, sensitivity, specificity, and other metrics of this test run, which we define in eTable 1 in Supplement 1.

To better understand our BoW models, we measured a word’s importance by the absolute value of coefficient weights of the L2-regularized logistic regression model. For our neural models, we used the Captum interpretability library for PyTorch^32^ to implement integrated gradients.^33^ This attribution method allows us to visualize what words in a document positively or negatively contribute to a prediction. While interpretable, the visualization is specific to words in an individual document; a word’s or phrase’s shown importance may be different in the context of a different document. To protect privacy, we anonymized the shown text by changing dates, names, and other identifying components, ensuring this did not change the interpretation.

## Results

### Patient and Document Selection

Of the 59 800 patients from BC Cancer, we excluded 2784 who were recorded as starting cancer care multiple times. Of these remaining 57 016 patients, 9391 did not have a consultation from a medical or radiation oncologist within 180 days following their cancer diagnosis. This left 47 625 patients fulfilling our inclusion and exclusion criteria; all had updated mortality data. This cohort consisted of 25 428 female patients (53.4%) and 22 197 male patients (46.6%) with a mean (SD) age of 64.9 (13.7) years (Table 1). We observed patients surviving a mean (SD) of 61.7 (40.3) months after diagnosis, and 59.9 (39.9) months after their initial oncologist consultation; these numbers are limited by the observation period, which was a minimum of 5 years. For our prediction targets, 41 447 (87.0%) survived 6 months, 31 143 (65.4%) survived 36 months, and 27 880 (58.5%) survived 60 months, calculated from the initial oncologist consultation.

**Table 1.** Characteristics of Patients in the Final Data Set^a^

Characteristic	Data (N = 47 625)
Sex	
Female	25 428 (53.4)
Male	22 197 (46.6)
Stage	
I	6505 (13.7)
II	8817 (18.5)
III	6227 (13.1)
IV	6287 (13.2)
Unknown	19 789 (41.6)
Age at diagnosis, mean (SD), y	64.9 (13.7)
Observed time survived, mean (SD), mo	
Since diagnosis^b^	61.7 (40.3)
Since document^b^^,^^c^	59.9 (39.9)
Time survived, mean (SD), mo	
Since diagnosis of those who died	27.0 (26.9)
Since document of those who died^c^	25.6 (26.6)
Between diagnosis and document generation^c^	1.34 (1.26)
Survived, mo	
6	41 447 (87.0)
36	31 143 (65.4)
60	27 880 (58.5)

^a^ Unless otherwise indicated, data are expressed as No. (%) of patients.

^b^ Indicates the number of months patients survived during the study’s observation period, which was at least 60 months.

^c^ Indicates the number of months survived since the initial oncologist consultation document used in this study was generated.

### Model Performance

In Table 2, we show how well our different NLP models predict whether patients will survive 5 years after their initial oncologist consultation when evaluated on a holdout test set. We see numerically similar performance between BoW, CNN, and LSTM, with BAC above 0.800 and AUC above 0.900. BERT has a lower performance across most metrics.

**Table 2.** Model Performance for Predicting 60-Month Survival After Patients’ Initial Oncologist Consultation Document Was Generated

Model	Accuracy	BAC	AUC	F1	Sensitivity	Specificity
BoW	0.823	0.835	0.915	0.856	0.798	0.871
CNN	0.828	0.837	0.918	0.862	0.809	0.866
LSTM	0.826	0.834	0.914	0.860	0.810	0.859
BERT	0.804	0.813	0.894	0.842	0.787	0.838

Abbreviations: AUC, receiver operating characteristics area under the curve; BAC, balanced accuracy; BERT, bidirectional encoder representations from transformers; BoW, bag-of-words; CNN, convolutional neural network; LSTM, long short-term memory.

### Performance by Survival Length

Table 3 compares the performance for our BoW model with one of our best neural models, CNN, when predicting the different survival lengths. On a holdout test set, BoW performed best for predicting 6-month survival, achieving a BAC of 0.856 (AUC 0.928); CNN had best performance for predicting 36-month survival, with a BAC of 0.842 (AUC, 0.918) and for 60-month survival, with a BAC of 0.837 (AUC, 0.918). We see similar performance between both models. Additional metrics, and performance of all models when predicting all durations, are described in eTable 2 in Supplement 1.

**Table 3.** Model Performance for Predicting the Given Numbers of Months After Patients’ Initial Oncologist Consultation Document Was Generated

Model^a^	Survival, mo	Accuracy	BAC	AUC	F1	Sensitivity	Specificity
BoW	6	0.849	0.856	0.928	0.907	0.847	0.866
CNN	6	0.843	0.853	0.926	0.903	0.839	0.867
BoW	36	0.837	0.837	0.915	0.872	0.836	0.838
CNN	36	0.844	0.842	0.918	0.878	0.848	0.836
BoW	60	0.823	0.835	0.915	0.856	0.798	0.871
CNN	60	0.828	0.837	0.918	0.862	0.809	0.866

Abbreviations: AUC, receiver operating characteristics area under the curve; BAC, balanced accuracy; BoW, bag-of-words; CNN, convolutional neural networks.

^a^ Comparison of the traditional model, BoW, with our best performing neural model, CNN.

### Interpretation

When observing the importance of tokens (words with endings removed), we see some similar tokens have top 10 importance for BoW models used to predict both 6- and 60-month survival (Table 4), but also some differences. For example, the token *palliat*, which can stem from the words *palliative* and *palliation*, is the most important feature in both. Different cancer locations or types are important for the 2 durations; breast and prostate are positive predictors for 6-month survival, while liver, glioblastoma, and lung are negative predictors for 60-month survival. The token *N0* is a top 10 positive predictor for 60-month survival, although we see no other TNM classification tokens in top 10 features for either survival duration.

**Table 4.** Top 10 Tokens Used by Bag-of-Words Models for 6- and 60-Month Survival Prediction^a^

Feature importance rank	Survival prediction			
	6 mo		60 mo	
	Token	Coefficient direction	Token	Coefficient direction
1	Palliat	Negative	Palliat	Negative
2	Poor	Negative	Metastat	Negative
3	Risk	Positive	Risk	Positive
4	Hospit	Negative	Liver	Negative
5	No	Positive	No	Positive
6	Unfortun	Negative	Glioblastoma	Negative
7	Prostat	Positive	Poor	Negative
8	Breast	Positive	Lung	Negative
9	Metastat	Negative	N0^b^	Positive
10	Stage	Positive	Whitehors	Positive

^a^ Feature importance was calculated using the absolute value of coefficient weights in these L2-regularized logistic regression models. Tokens are words in which the word endings have been removed for processing.

^b^ Note that N0 refers to a patient without disease in lymph nodes in TNM cancer staging.

eFigures 1 and 2 in Supplement 1 show the importance of words in a patient’s document for our CNN models, which correctly predict that this patient survives 6 but not 60 months. We again see similarities and differences between the models developed to predict surviving both lengths. For example, the patient’s age had negative importance when predicting whether they would survive 60 months, but not 6 months. Positive aspects of the surgery and pathology, such as having clear margins and no lymphatic or vascular involvement, predicted positively in both models, but more so for the 6-month survival prediction. Medical comorbidities such as congestive heart failure and Barrett esophagus were negative predictors for 60-month survival, but less so in predicting 6-month survival.

## Discussion

In this study, we trained and evaluated NLP models using both traditional and neural models to predict whether a patient with cancer will survive 6, 36, and 60 months using only their initial oncologist consultation document and no other data. The performance of our models, when evaluated on a never-before-seen internal holdout set, achieved accuracy, BAC, and AUC over 0.800, with our best models achieving AUC over 0.900. This performance was similar to or better than prior work seeking to predict survival of patients with cancer,^5,8,16,34,35,36^ despite using data that were more generalizable and readily available. Prior studies have predicted cancer survival for specific tumor sites such as breast or lung, or with the use of structured data such as processed clinical and genetic characteristics.

Our results suggest it is possible to predict the survival of patients with cancer without having to construct structured data sets, or limiting the predictions to specific types or locations of cancer. The availability of structured data may vary. Given the widespread availability of initial oncologist consultation documents, this opens up the possibility of more easily training and using such models across cancer types at different cancer centers.

To help our models be generalizable, we did minimal text processing. We did not extract features from the text to be used by our models, instead providing text directly. The documents in our data set were generated by oncologists practicing at 6 centers located in geographically varied regions without set templates or formatting requirements. Future work will be needed to externally validate our models on documents generated in other jurisdictions.

The performance of our CNN, LSTM, and BoW models was numerically similar. BERT’s inferior performance, despite its more deeply connected network, may be due to its limitation of only being able to use the first 512 tokens. The lack of clear performance gain with neural models compared with the traditional BoW model is surprising, but in line with some prior work.^37,38^ This may suggest survival prediction is largely dependent on the presence of words, compared with understanding how words relate to each other in a document.

We interpreted our neural models using integrated gradients and our BoW models using coefficient values. Both methods have their limitations. Feature correlation impacts L2-regularized coefficients, while integrated gradients show word importance based on their specific context. However, our results generally supported that our models used generalizable, interpretable portions of text to make their predictions, consistent with known mortality risk and protective factors.^39,40^ For example, palliative care is often accessed in the last months of a patient’s life, so it is unsurprising that an oncologist mentioning palliative care would support that a patient may not survive 6 months. The 60-month BoW model included N0, that a patient has no known spread to lymph nodes, but not the 6-month BoW model. Nodal status could be expected to be less relevant in the shorter time. We found that the neural model weighed clinically relevant data such as age, positive outcomes from a surgery, and comorbidities differently when predicting 6- or 60-month survival.

There are multiple avenues for further development to build on our results. While some related work found adding structured data led to only a small, likely not clinically significant improvement in performance,^15^ it may still be worthwhile investigating the addition of structured data. Future work could improve on our results by using different models or configurations, including those both more and less complex. Hardware advancement will allow more complex versions of our models, and there are new transformer models such as Longformer^41^ and BigBird^42^ that can use more text. Neural models can especially benefit from training on very large amounts of data. Future work could use our methodology to train models using widely available oncologist consultation documents from multiple jurisdictions or fine-tune our models by further training them with a relatively small number of a different jurisdiction’s documents. Future research could also adapt our models to update survival prediction after the initial consultation, such as by using progress notes. Further performance improvements and external validation may allow clinicians to use this methodology for improving care, such as better facilitating palliative resources with short-term survival predictions or using long-term predictions to consider more intense initial treatment.

### Limitations

This study has some limitations. We took steps to ensure the validity of our models, including the use of a holdout test set, and eliminating location-specific text that was automatically added to documents, but we did not externally validate our models. Our work calculated survival from the first oncologist consultation document, as opposed to from diagnosis, as is common. This was done because the time from diagnosis to consultation can vary from a few weeks to a few months at BC Cancer if patients first received treatment such as surgery from outside clinicians, or initial workup from family physicians. We also note our data set consisted of patients first seen between 2011 and 2016, so we could establish 5-year survival. Given the rapid advancement of cancer treatment, accuracy may be limited for patients starting treatment presently. Finally, we did not compare our models’ performance with that of oncologists, as has been done in previous work.

## Conclusions

In this prognostic study of 47 625 patients with cancer, we trained and evaluated traditional and neural models to predict whether patients will survive 6, 36, or 60 months based on their initial oncologist consultation. We evaluated our model on an internal holdout set of data, and found our best models achieved accuracy and BAC above 0.800 and AUC above 0.900. This performance was comparable with or superior to that of prior work, which has predicted survival only for specific types of cancer or used data that are more difficult to obtain.

Our findings suggest that a clinically useful survival prediction model for patients with general cancer may be possible without needing specific models for different cancer types and by using data that are readily accessible without complex data processing or data mining. These results still require external validation and have multiple avenues for further improvement; regardless, they suggest that this methodology may one day assist in care by helping patients with cancer and their treatment team by providing an individualized expectation of survival.

## Notes

Supplement 1. **eMethods.** Obtaining Data, Text Processing, Language Models Used, Hardware Used, and Implementation **eTable 1.** Definition of Evaluation Metrics Reported in This Work **eTable 2.** Performance of All Models When Predicting Surviving the Given Number of Months, With Extended Metrics **eFigure 1.** Visualizing Word Importance of CNN Models Used to Predict 6- and 60-Month Survival **eFigure 2.** Visualizing Word Importance of CNN Models Used to Predict 6- and 60-Month Survival, Adapted for Color Blindness
eReferences

Supplement 2.
Data Sharing Statement
