# Supplementary material for: Predicting the Survival of Patients With Cancer From Their Initial Oncology Consultation Document Using Natural Language Processing
Source: JAMA Netw Open. 2023 Feb 27;6(2):e230813. doi: 10.1001/jamanetworkopen.2023.0813 (PMC9972192; doi:10.1001/jamanetworkopen.2023.0813)
Supplement: Supplement 1. — eMethods. Obtaining Data, Text Processing, Language Models Used, Hardware Used, and Implementation eTable 1. Definition of Evaluation Metrics Reported in This Work eTable 2. Performance of All Models When Predicting Surviving the Given Number of Months, With Extended Metrics eFigure 1. Visualizing Word Importance of CNN Models Used to Predict 6- and 60-Month Survival eFigure 2. Visualizing Word Importance of CNN Models Used to Predict 6- and 60-Month Survival, Adapted for Color Blindness eReferences [file jamanetwopen-e230813-s001.pdf]

Supplementary Online Content

Nunez JJ, Leung B, Ho C, Bates AT, Ng RT. Predicting the survival of patients with cancer from their initial oncology consultation document using natural language processing. *JAMA Netw Open*. 2023;6(2):e230813. doi:10.1001/jamanetworkopen.2023.0813

eMethods. Obtaining Data, Text Processing, Language Models Used, Hardware Used, and Implementation
eTable 1. Definition of Evaluation Metrics Reported in This Work
eTable 2. Performance of All Models When Predicting Surviving the Given Number of Months, With Extended Metrics
eFigure 1. Visualizing Word Importance of CNN Models Used to Predict 6- and 60-Month Survival
eFigure 2. Visualizing Word Importance of CNN Models Used to Predict 6- and 60-Month Survival, Adapted for Color Blindness
eReferences

This supplementary material has been provided by the authors to give readers additional information about their work.

© 2023 Nunez JJ et al. *JAMA Network Open*.

eMethods. Obtaining Data, Text Processing, Language Models Used, Hardware Used, and Implementation

Obtaining Data

Unstructured text documents were provided as extracted Microsoft Word documents from BC Cancer electronic health records, along with some structured data including diagnosis date, age, and cancer site at diagnosis. Additional structured data, specifically death dates, were obtained from the BC Vital Statistics by BC Cancer data stewards, and linked to our dataset. We also received metadata on extracted documents, including the medical speciality that generated the document, and the document type. We used this metadata in our document selection, excluding documents that were not consultation documents such as progress notes.

Text Processing

We replaced question and exclamation marks with periods, all spaces with single spaces, and we converted characters that were not alphanumeric, parenthesis, apostrophes, or punctuation with spaces. We removed automatically added text at the beginning and end of the documents that contained information such as identifying information, dates, and the names of the dictating providers and who they sent the documents to; these string patterns are available in the project's Github repository.

Language Models Used

Bag-of-word (BoW) models have a simple understanding of documents, simply counting the frequency that certain words occur in a document[1]. These frequencies then form a vector, which can be used by traditional machine learning algorithms. We implemented BoW with common choices: L2-regularized logistic regression, and term frequency-inverse document frequency weighting[2]. We used a C of 1, corresponding to the inverse of the lambda regularization factor, and a vector length of 5000, tuning these hyperparameters empirically.

When used in natural language processing (NLP), convolutional neural networks (CNN)'s use convolutions of a small number of adjacent words[1]. They then understand a document based on combinations of these small groupings, allowing a reduction of the feature space, but distant word relationships are still considered. We based our CNN models on those developed for general and medical document classification[3–5]. We based our hyperparameters off these works, with some additional fine-tuning, using 300-length word vectors, window lengths of 3,4 and 5 tokens, 500 output channels, and a dropout rate of 0.8 with a learning rate of 0.00005.

Long short-term memory (LSTM) models are a type of recurrent neural network that understands a document one-word at a time, changing the prediction at every step[1]. LSTM have memory cells that allow the model to better consider what words occurred in other parts of the document. We based our LSTM on previous models developed using regularization to avoid overfitting[6]. Again fine-tuning previously used hyperparameters, we used word embeddings with length 300, a hidden unit dimension of 512, general dropout of 0.5, word embedding dropout of 0.1, weight dropout of 0.2, and learning rate of 0.00005.

The bidirectional encoder representation from transformers (BERT) model was developed by Devlin et al[7] to allow a deep, bidirectional understanding of language. Using state-of-the-art computational resources, they developed a transformer model which allows all pieces of text in a document to be considered at once. This model uses attention to focus on useful relationships. This model can then be fine-tuned to accomplish other tasks, such as our binary survival classification. While allowing all words to be considered with respect to how they relate to each other, these models are limited in only being able to utilize 512 tokens, which represent an entire or part of a word. We used the general English BERT model, *bert-base-uncased*, a weight dropout of 0.001, and a learning rate of 0.00005.

© 2023 Nunez JJ et al. *JAMA Network Open*.

Hardware Used

All computation for this work was performed on a virtual installation of Windows Server 2012 R2, with an eight processor Intel Xeon 8160 CPU, and 16 GB of RAM. We had access to a shared GPU through a NVIDIA GRID V100D-16Q, with 16 GB of VRAM allocated to our virtualisation. We ran the neural models on the virtual GPU, and BoW models on the CPU.

Implementation

We implemented our BoW model with the scikit-learn library[8], while we used PyTorch[9] and PyTorch Lightning[10] for our neural models, the last being used to reduce boilerplate code. We used the Pandas data processing library [11] for data processing, target generation, and analysis.

As the proportion of patients surviving to our cut-offs varied (58.5 - 87.0%), we used loss weighting to account for this class imbalance. We adjusted our binary cross entropy loss by the inverse of the relative class proportion.

Once published, code and trained models are available on the public GitHub repository https://github.com/jjnunez11/scar_nlp_survival.

© 2023 Nunez JJ et al. *JAMA Network Open*.

eTable 1. Definition of Evaluation Metrics Reported in This Work

**Metric**	**Definition**
Accuracy	$\frac{TP+TN}{TP+TN+FP+FN}$
Balanced Accuracy	$\frac{\frac{TP}{TP+FN}+\frac{TN}{TN+FP}}{2}$
Recall/Sensitivity	$\frac{TP}{TP+FN}$
Specificity	$\frac{TN}{TN+FP}$
Precision/Positive Predictive Value	$\frac{TP}{TP+FP}$
Negative Predictive Value	$\frac{TN}{TN+FN}$
F1	$\frac{2TP}{2TP+FP+FN}$
AUC	Integral of the receiver-operator-curve, formed by plotting sensitivity against 1-specificity at various detection thresholds.

Abbreviations: TP, true positive; TF, true negative; FP, false positive; FN, false negative; AUC, receiver-operator curve area-under-curve.

© 2023 Nunez JJ et al. *JAMA Network Open*.

eTable 2. Performance of All Models When Predicting Surviving the Given Number of Months, With Extended Metrics

Months	Model	Acc.	BAC	AUC	F1	Rec.	Prec.	Spec.	PPV	NPV	TP	TN	FP	FN
6	BoW	0.849	0.856	0.928	0.907	0.847	0.977	0.866	0.977	0.455	7067	1067	165	1278
6	CNN	0.843	0.853	0.926	0.903	0.839	0.977	0.867	0.977	0.443	7004	1068	164	1341
6	LSTM	0.799	0.847	0.923	0.872	0.783	0.984	0.912	0.984	0.382	6532	1123	109	1813
6	BERT	0.859	0.815	0.906	0.915	0.874	0.960	0.756	0.960	0.470	7294	932	300	1051
36	BoW	0.837	0.837	0.915	0.872	0.836	0.910	0.838	0.910	0.724	5298	2718	525	1036
36	CNN	0.844	0.842	0.918	0.878	0.848	0.910	0.836	0.910	0.738	5369	2712	531	965
36	LSTM	0.830	0.836	0.914	0.864	0.817	0.917	0.856	0.917	0.706	5176	2775	468	1158
36	BERT	0.799	0.815	0.901	0.834	0.765	0.917	0.866	0.917	0.654	4847	2807	436	1487
60	BoW	0.823	0.835	0.915	0.856	0.798	0.923	0.871	0.923	0.688	5056	2824	419	1278
60	CNN	0.828	0.837	0.918	0.862	0.809	0.922	0.866	0.922	0.699	5124	2808	435	1210
60	LSTM	0.826	0.834	0.914	0.860	0.810	0.918	0.859	0.918	0.698	5128	2785	458	1206
60	BERT	0.804	0.813	0.894	0.842	0.787	0.905	0.838	0.905	0.668	4985	2719	524	1349

Abbreviations**:** Acc.: accuracy, BAC: balanced accuracy, AUC: receiver-operator-curve area-under-curve, Rec.: Recall/Sensitivity, Prec.: Precision, Spec.: Specificity, PPV: positive predictive value, NPV: negative predictive value, TP: true positives, TN: true negatives, FP: false positives, FN: false negatives, BoW: bag-of-words, CNN: convolutional neural networks, LSTM: long short-term memory, BERT: bidirectional encoder representations from transformers.

© 2023 Nunez JJ et al. *JAMA Network Open*.

eFigure 1. Visualizing Word Importance of CNN Models Used to Predict 6- and 60-Month Survival

60-month Survival

01 consulting service medical oncology consultation requested by revised report please destroy previous report . (april 5, 2016) . reason addendum added joseph is an 83 year old male who presented to the emergency with hematochezia . he had a colonoscopy on february 18th . it was advanced to the cecum . at the cecum , there was a large villous growth . in the distal ascending colon , there was evidence of a possible invasive adenocarcinoma . his anastomosis from his previous surgery at the rectosigmoid junction , showed no evidence of recurrence . pathology of the mid ascending colon mass showed invasive colonic adenocarcinoma . the cecal biopsy showed tubular adenoma with microscopic focus of high grade dysplasia . ces february 18th was 5 . 7 , normal less than 5 . 0 . ct chest , abdomen and pelvis from february 21 , showed no evidence of malignancy , on february 28th , the patient had laproscopic right hemicolectomy and laparoscopic lysis of adhesions . pathology from his surgery showed an infiltrative well differentiated colonic adenocarcinoma based within the ascending colon infiltrating focally into the muscularis propria , but did not involve full thickness of muscularis propria . there is no lymphatic or vascular invasion . resection margins were negative . the 11 lymph nodes were benign . there is also a cecal tubulovillous adenoma with high surface grade dysplasia . there was also a background of 2 separate tubular adenomata without areas of high grade dysplasia or malignancy . no significant problems since surgery . weight is stable since discharge . his energy is back to where it was 6 months ago . he can dress and bathe himself . he can do cooking but he cannot do cleaning . i would put him at a good ecog 2 . since discharge , he has been using oxygen when he exerts himself and he has also since discharge newly using a walker . he has 1 formed bowel movement per day . he has no headache , no nausea , no vomiting . no visual problems . no new lumps or bumps . no bone pain or bone tenderness . chronic shortness of breath on exertion , not significantly changed . no chest pain , no cough , no abdominal pain . no melena and no hematochezia . history of past health shows a sick sinus syndrome and he has a pacemaker in . he has an lv ejection of 45 and previous episodes of congestive heart failure . echo shows pulmonary hypertension , mitral stenosis , mitral regurgitation and tricuspid regurgitation . he had a positive mibi test in 2006 . he had a previous gi bleed in april 2010 . he has a history of nephrolithiasis which required intervention , barrett ' s esophagus , hypothyroidism , dyslipidemia . he

6-month Survival

01 consulting service medical oncology consultation requested by revised report please destroy previous report . (april 5, 2016) . reason addendum added joseph is an 83 year old male who presented to the emergency with hematochezia . he had a colonoscopy on february 18th . it was advanced to the cecum . at the cecum , there was a large villous growth . in the distal ascending colon , there was evidence of a possible invasive adenocarcinoma . his anastomosis from his previous surgery at the rectosigmoid junction , showed no evidence of recurrence . pathology of the mid ascending colon mass showed invasive colonic adenocarcinoma . the cecal biopsy showed tubular adenoma with microscopic focus of high grade dysplasia . ces february 18th was 5 . 7 , normal less than 5 . 0 . ct chest , abdomen and pelvis from february 21 , showed no evidence of malignancy , on february 28th , the patient had laproscopic right hemicolectomy and laparoscopic lysis of adhesions . pathology from his surgery showed an infiltrative well differentiated colonic adenocarcinoma based within the ascending colon infiltrating focally into the muscularis propria , but did not involve full thickness of muscularis propria . there is no lymphatic or vascular invasion . resection margins were negative . the 11 lymph nodes were benign . there is also a cecal tubulovillous adenoma with high surface grade dysplasia . there was also a background of 2 separate tubular adenomata without areas of high grade dysplasia or malignancy . no significant problems since surgery . weight is stable since discharge . his energy is back to where it was 6 months ago . he can dress and bathe himself . he can do cooking but he cannot do cleaning . i would put him at a good ecog 2 . since discharge , he has been using oxygen when he exerts himself and he has also since discharge newly using a walker . he has 1 formed bowel movement per day . he has no headache , no nausea , no vomiting . no visual problems . no new lumps or bumps . no bone pain or bone tenderness . chronic shortness of breath on exertion , not significantly changed . no chest pain , no cough , no abdominal pain . no melena and no hematochezia . history of past health shows a sick sinus syndrome and he has a pacemaker in . he has an lv ejection of 45 and previous episodes of congestive heart failure . echo shows pulmonary hypertension , mitral stenosis , mitral regurgitation and tricuspid regurgitation . he had a positive mibi test in 2006 . he had a previous gi bleed in april 2010 . he has a history of nephrolithiasis which required intervention , barrett ' s esophagus , hypothyroidism , dyslipidemia . he

Abbreviations: CNN, convolutional neural network.

© 2023 Nunez JJ et al. *JAMA Network Open*.

We show a visualization of word importance using integrated gradients for convolutional neural network models predicting 60-month and 6-month survival. We used anonymized text from a patient that lived 24 months. The darker the green background of a token the more it predicted survival in this context, while the redder, the more it was a negative predictor. We use the default Captum colours here, see eFigure 2 in the Supplement for a version adapted for colour-blindness.

© 2023 Nunez JJ et al. *JAMA Network Open*.

eFigure 2. Visualizing Word Importance of CNN Models Used to Predict 6- and 60-Month Survival, Adapted for Color Blindness

60-month Survival

01 consulting service medical oncology consultation requested by revised report please destroy previous report . (april 5, 2016) . reason addendum added joseph is an 83 year old male who presented to the emergency with hematochezia . he had a colonoscopy on february 18th . it was advanced to the cecum . at the cecum , there was a large villous growth . in the distal ascending colon , there was evidence of a possible invasive adenocarcinoma . his anastomosis from his previous surgery at the rectosigmoid junction , showed no evidence of recurrence . pathology of the mid ascending colon mass showed invasive colonic adenocarcinoma . the cecal biopsy showed tubular adenoma with microscopic focus of high grade dysplasia . ces february 18th was 5 . 7 , normal less than 5 . 0 . ct chest , abdomen and pelvis from february 21 , showed no evidence of malignancy , on february 28th , the patient had laproscopic right hemicolectomy and laparoscopic lysis of adhesions . pathology from his surgery showed an infiltrative well differentiated colonic adenocarcinoma based within the ascending colon infiltrating focally into the muscularis propria , but did not involve full thickness of muscularis propria . there is no lymphatic or vascular invasion . resection margins were negative . the 11 lymph nodes were benign . there is also a cecal tubulovillous adenoma with high surface grade dysplasia . there was also a background of 2 separate tubular adenomata without areas of high grade dysplasia or malignancy . no significant problems since surgery . weight is stable since discharge . his energy is back to where it was 6 months ago . he can dress and bathe himself . he can do cooking but he cannot do cleaning . i would put him at a good ecog 2 . since discharge , he has been using oxygen when he exerts himself and he has also since discharge newly using a walker . he has 1 formed bowel movement per day . he has no headache , no nausea , no vomiting . no visual problems . no new lumps or bumps . no bone pain or bone tenderness . chronic shortness of breath on exertion , not significantly changed . no chest pain , no cough , no abdominal pain . no melena and no hematochezia . history of past health shows a sick sinus syndrome and he has a pacemaker in . he has an lv ejection of 45 and previous episodes of congestive heart failure . echo shows pulmonary hypertension , mitral stenosis , mitral regurgitation and tricuspid regurgitation . he had a positive mibi test in 2006 . he had a previous gi bleed in april 2010 . he has a history of nephrolithiasis which required intervention , barrett ' s esophagus , hypothyroidism , dyslipidemia . he

6-month Survival

01 consulting service medical oncology consultation requested by revised report please destroy previous report . (april 5, 2016) . reason addendum added joseph is an 83 year old male who presented to the emergency with hematochezia . he had a colonoscopy on february 18th . it was advanced to the cecum . at the cecum , there was a large villous growth . in the distal ascending colon , there was evidence of a possible invasive adenocarcinoma . his anastomosis from his previous surgery at the rectosigmoid junction , showed no evidence of recurrence . pathology of the mid ascending colon mass showed invasive colonic adenocarcinoma . the cecal biopsy showed tubular adenoma with microscopic focus of high grade dysplasia . ces february 18th was 5 . 7 , normal less than 5 . 0 . ct chest , abdomen and pelvis from february 21 , showed no evidence of malignancy , on february 28th , the patient had laproscopic right hemicolectomy and laparoscopic lysis of adhesions . pathology from his surgery showed an infiltrative well differentiated colonic adenocarcinoma based within the ascending colon infiltrating focally into the muscularis propria , but did not involve full thickness of muscularis propria . there is no lymphatic or vascular invasion . resection margins were negative . the 11 lymph nodes were benign . there is also a cecal tubulovillous adenoma with high surface grade dysplasia . there was also a background of 2 separate tubular adenomata without areas of high grade dysplasia or malignancy . no significant problems since surgery . weight is stable since discharge . his energy is back to where it was 6 months ago . he can dress and bathe himself . he can do cooking but he cannot do cleaning . i would put him at a good ecog 2 . since discharge , he has been using oxygen when he exerts himself and he has also since discharge newly using a walker . he has 1 formed bowel movement per day . he has no headache , no nausea , no vomiting . no visual problems . no new lumps or bumps . no bone pain or bone tenderness . chronic shortness of breath on exertion , not significantly changed . no chest pain , no cough , no abdominal pain . no melena and no hematochezia . history of past health shows a sick sinus syndrome and he has a pacemaker in . he has an lv ejection of 45 and previous episodes of congestive heart failure . echo shows pulmonary hypertension , mitral stenosis , mitral regurgitation and tricuspid regurgitation . he had a positive mibi test in 2006 . he had a previous gi bleed in april 2010 . he has a history of nephrolithiasis which required intervention , barrett ' s esophagus , hypothyroidism , dyslipidemia . he

Abbreviations: CNN: convolutional neural network.

© 2023 Nunez JJ et al. *JAMA Network Open*.

We show a visualization of word importance using integrated gradients for convolutional neural network models predicting 60-month and 6-month survival. We used anonymized text from a patient that lived 24 months. The purple shading, as around “83 year old” in the 60-month survival text, corresponds to the model finding this a negative predictor of survival. Green shading, as around “tubular adenoma” in the 60-month survival excerpt, corresponds to the model using this as a positive predictor. A darker shade corresponds to more importance.

eReferences

1. Zhang A, Lipton ZC, Li M, Smola AJ. Dive into Deep Learning. *ArXiv210611342 Cs*. Published online July 26, 2021. Accessed February 20, 2022. http://arxiv.org/abs/2106.11342
2. Manning C, Raghavan P, Schuetze H. *Introduction to Information Retrieval*. Cambridge University Press; 2009.
3. Kim Y. Convolutional Neural Networks for Sentence Classification. In: *Proceedings of the 2014 Conference on Empirical Methods in Natural Language Processing (EMNLP)*. Association for Computational Linguistics; 2014:1746-1751. doi:10.3115/v1/D14-1181
4. Rios A, Kavuluru R. Convolutional neural networks for biomedical text classification: application in indexing biomedical articles. In: *Proceedings of the 6th ACM Conference on Bioinformatics, Computational Biology and Health Informatics*. ACM; 2015:258-267. doi:10.1145/2808719.2808746
5. Rios A, Kavuluru R. Ordinal Convolutional Neural Networks for Predicting RDoC Positive Valence Psychiatric Symptom Severity Scores. *J Biomed Inform*. 2017;75 Suppl:S85-S93. doi:10.1016/j.jbi.2017.05.008
6. Adhikari A, Ram A, Tang R, Lin J. Rethinking Complex Neural Network Architectures for Document Classification. In: *Proceedings of the 2019 Conference of the North*. Association for Computational Linguistics; 2019:4046-4051. doi:10.18653/v1/N19-1408
7. Devlin J, Chang MW, Lee K, Toutanova K. BERT: Pre-training of Deep Bidirectional Transformers for Language Understanding. *ArXiv181004805 Cs*. Published online May 24, 2019. Accessed February 20, 2022. http://arxiv.org/abs/1810.04805
8. Pedregosa F, Varoquaux G, Gramfort A, et al. Scikit-learn: Machine Learning in Python. *J Mach Learn Res*. 2011;12(Oct):2825-2830.
9. Paszke A, Gross S, Massa F, et al. PyTorch: An Imperative Style, High-Performance Deep Learning Library. In: *Advances in Neural Information Processing Systems*. Vol 32. Curran Associates, Inc.; 2019. Accessed February 19, 2022. https://papers.nips.cc/paper/2019/hash/bdbca288fee7f92f2bfa9f7012727740-Abstract.html
10. William Falcon, The PyTorch Lightning Team. PyTorch Lightning.
11. McKinney W. pandas: a Foundational Python Library for Data Analysis and Statistics. :9.
12. Kokhlikyan N, Miglani V, Martin M, et al. Captum: A unified and generic model interpretability library for PyTorch. *ArXiv200907896 Cs Stat*. Published online September 16, 2020. Accessed February 19, 2022. http://arxiv.org/abs/2009.07896

© 2023 Nunez JJ et al. *JAMA Network Open*.
