# Supplementary material for: Predicting the Survival of Patients With Cancer From Their Initial Oncology Consultation Document Using Natural Language Processing
Source: JAMA Netw Open. 2023 Feb 27;6(2):e230813. doi: 10.1001/jamanetworkopen.2023.0813 (PMC9972192; doi:10.1001/jamanetworkopen.2023.0813)
Supplement: Supplement 2. — Data Sharing Statement [file jamanetwopen-e230813-s002.pdf]

Data Sharing Statement

Nunez. Predicting the Survival of Patients With Cancer From Their Initial Oncology Consultation Document Using Natural Language Processing. *JAMA Netw Open*. Published February 27, 2023. doi:10.1001/jamanetworkopen.2023.0813

Data
Data available: No

Additional Information
Explanation for why data not available: The medical records and documents used in this dataset are personally identifying, and cannot be suitably de-identified at this time. We are not able to share these data currently, though long-term discussions are taking place about one day making this dataset available. The computer code and trained models will be made publicly available upon publication on the github repository https://github.com/jjnunez11/scar_nlp_survival
